# Predictive Value of Grooming Behavior for Development of Dermatitis in Selectively Bred P Rats as a Model of Trichotillomania Hair Pulling Disorder

**DOI:** 10.3390/vetsci9020089

**Published:** 2022-02-18

**Authors:** Debra Hickman, Anjali Prakash, Richard Bell

**Affiliations:** 1 Laboratory Animal Resource Center, School of Medicine, Indiana University, Indianapolis, IN 46202, USA; prakasha@indiana.edu; 2 Department of Comparative Pathobiology, College of Veterinary Medicine, Purdue University, West Lafayette, IN 47907, USA; 3 Department of Psychiatry, School of Medicine, Indiana University, Indianapolis, IN 46202, USA; ribell@iupui.edu

**Keywords:** animal model, trichotillomania, hair-pulling, body-focused repetitive disorder

## Abstract

Trichotillomania (TTM) is a body-focused repetitive disorder affecting as much as 0.5 to 2% of the population, with women four times more likely to be affected than men. This disorder causes impairment in daily function and significant distress. A potential animal model for this disorder is the inbred C57BL/6J mouse which displays clinical signs and behavioral characteristics similar to those described for people affected by this disorder. Because alcohol-preferring P rats also display similar clinical signs and behavioral characteristics, it was hypothesized that this selectively bred stock could be an additional animal model. In this study, 112 female P rats were recorded on digital media for 15 min after being sprayed with a mist of water and assessed for grooming patterns—oral, manual, and scratching. Significant elevations in scratching and oral grooming behavior were predictive of the future development of skin lesions. These findings suggest that P rats may be an additional model to study TTM, with the advantage of increased genetic variation (i.e., non-inbred) which mirrors the human population. The use of this model may help to identify preventative and therapeutic interventions for humans and other animals with similar body-focused repetitive disorders.

## 1. Introduction

Trichotillomania (TTM, “hair-pulling disorder”) is defined by the American Psychiatric Association as an obsessive compulsive disorder (OCD) [1]. People who are affected by TTM pull their hair out, creating bald patches, resulting in exposed skin and tissue, with the disorder being typically diagnosed in childhood or adolescence. The affected areas can include anywhere there is hair, with the scalp, eyebrows, eyelashes, pubic area, and beard representing the most common locations [1]. The true incidence rate is not known due to the social impairment induced by the condition, but it is estimated as high as 3% of the population [2]. Females are affected more frequently than males, with females representing 92.5% of the adult patients presenting to a TTM clinic, though it has been hypothesized that men may be better able to hide the behavior through interventions such as shaving the head. The diagnostic criteria for TTM include the recurrent pulling out of one’s hair that results in noticeable hair loss, repeated attempts to decrease or stop the hair-pulling behavior, and clinically significant distress or impairment in social, occupational, or other areas of functioning [1]. Additionally, the condition must not be accounted for by another mental disorder or be due to a general medical condition (e.g., dermatologic condition). Often, these individuals exhibit more than one body-focused repetitive disorder, including nail biting, skin picking, thumb sucking, knuckle cracking, or nose picking [2,3,4,5,6,7,8].

Because the disorder often results in chastisement or teasing, individuals work to hide the disorder and frequently develop low self-esteem. Individuals also suffer from other psychiatric disorders, such as depression (57%), generalized anxiety (27%), simple phobia (19%), alcohol abuse (19%), obsessive compulsive disorder (13%), social phobia (11%), and eating disorders (11%) [4,8,9,10,11,12,13,14,15]. In addition to social impairment concerns, people suffering from this disorder may also ingest the hair, leading to secondary complications, such as trichobezoars and intestinal obstruction [12].

Treatment of TTM is challenging with behavioral, dietary, and pharmaceutical interventions having limited efficacy [8,9,16,17,18,19,20,21]. Human trials have the limitation of a high amount of genetic and environmental variation, in addition to a long lifespan and relatively low incidence of diagnosed candidates for clinical trials. The development of an animal model that mimics the clinical presentation of humans would be valuable by providing an experimental condition with fewer confounding factors (e.g., genetics and/or environment), leading to a better understanding of the underlying pathology and developing strategies to treat or prevent this disorder. There are a number of different animals that engage in similar body-focused repetitive disorders, such as feather picking in birds [22,23,24,25,26,27,28,29,30,31], psychogenic alopecia in cats [32,33,34,35,36] and nonhuman primates [37,38,39], acral lick dermatitis in dogs [35,40,41,42], tail and ear biting in swine [43,44,45], and flank biting in horses [46,47,48].

The C57BL/6J mouse, an inbred strain, has a common clinical presentation of “barbering” (hair loss) and idiopathic necrotizing dermatitis, especially prevalent in stressful situations [49,50,51,52,53]. Although hair loss has been noted as a precursor to idiopathic dermatitis, the phenomenon of barbering has historically been attributed to behavioral stressors in social dominance interactions [51,54]. However, recent work suggests that barbering is more appropriately diagnosed as a stereotypic behavior and can be self-directed [50,51,53]. Building on these assessments, the C57BL/6J mouse has been proposed as a potential model for TTM and a similar disorder, dermatotillomania [50]. These authors determined that they could predict the likelihood of developing hair loss and skin lesions through a simple behavioral test that was performed before the mouse exhibited clinical symptoms. They found that if they gently sprayed the mouse with water and observed its grooming pattern for 15 min, the proportion of time that the mouse spent scratching during grooming was predictive of an increased chance of developing hair loss and skin lesions in the future with an odds ratio of 1.20 [50]. This inbred mouse model has already been used to evaluate the effect of diet on the development of TTM and dermatotillomania [50].

**Figure 1:**
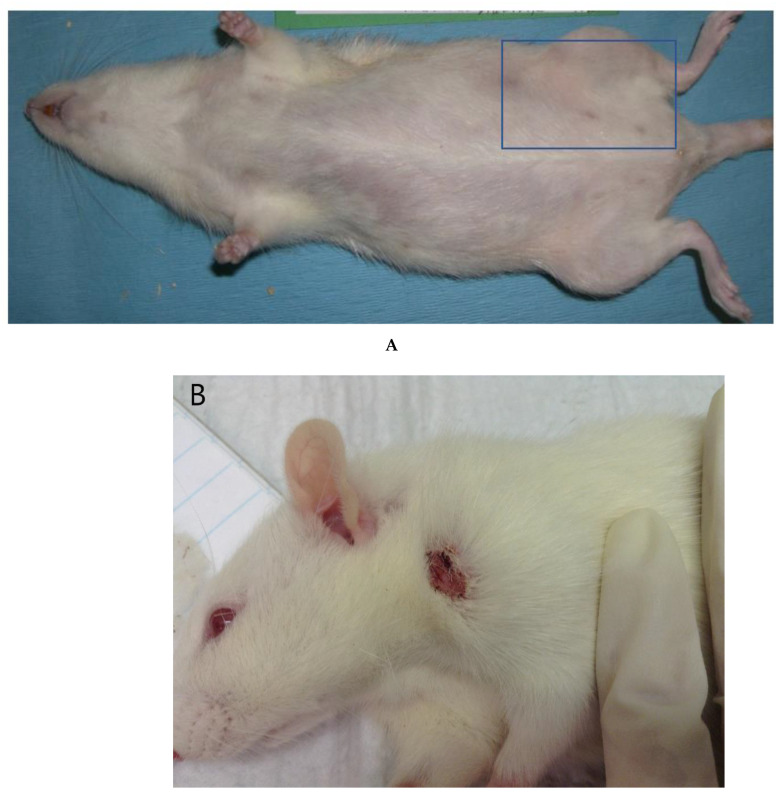
Examples of clinical presentations of P rats with (**A**) hair loss and (**B**) dermatitis. Note that the hair loss is most extensive in the inguinal region (box), extending down the hindlimbs, though there is thinning over the entire ventrum (**A**). The development of dermatitis (**B**) is typically around the head and neck region.

In the Indiana University (IU) breeding colonies that support the production of the alcohol-preferring “P” rat, we have also noticed an exhibition of a clinical predisposition to hair loss and ulcerative dermatitis. The clinical presentation is variable, with hair loss occurring over the entire body, especially on the ventrum, neck, axillary, and inguinal regions (Figure 1A) as well as dermatitis lesions generally focused around the neck and ears (Figure 1B). Because this rat stock is not inbred [55,56], the use of this animal model has the potential to better approximate the human condition, with the genetic diversity of these populations. Although inbred mouse models and tightly controlled environmental conditions have been used extensively for the characterization and understanding of human diseases, the current debate has suggested that this could be contributing to the issues noted when translating findings from rodent models to humans [57,58,59]. For example, scientists evaluating an outbred model for vaccine development and testing noted that reliance on inbred mouse strains could lead to scientists being misled regarding the effectiveness of various vaccines in the diverse human population [60]. Identification of animal models with more genetic diversity could be invaluable models for human diseases.

The C57BL/6J mouse and the P rat are currently used as models for addiction studies [61,62,63,64,65,66]. They also consistently test as more anxious in assays to measure depression and anxiety when compared to other rodent models [56]. The comorbidity of these conditions with the expression of TTM is similar to the presentation seen in humans, further supporting the potential applicability of these rodents as animal models of TTM.

In this study, the spray test and subsequent grooming behavior assessment were used to determine if there was a behavioral pattern that was predictive of future development of hair loss and skin lesions in the P rat, to determine the applicability of this rat as a potential animal model of TTM.

## 2. Materials and Methods

### 2.1. Ethical Statement

All procedures were reviewed and approved by the IU School of Medicine IACUC prior to the initiation of the project. The program is accredited by AAALAC International and compliant with all applicable federal regulations.

### 2.2. Animals

The alcohol-preferring P rat was the line of rat used in this study. The P rat was developed through bidirectional (vs the alcohol-non-preferring NP rat) mass selection from a closed-colony of Wistar rats at the Walter Reed Army Hospital [66]. P rats were transferred to the Indiana University School of Medicine, and they have been maintained by the Indiana Alcohol Research Center, Indianapolis, IN, USA, since then. Two criteria determined the alcohol-preferring P phenotype. First, the animal preferred an unadulterated 10% ethanol solution over water by a ratio of at least 2:1; and second, the animals consumed more than 5 g of ethanol/kg body weight/day [55,65,66]. Five g/kg/day is equivalent to a 70 kg person consuming approximately a fifth of 90-proof whiskey per day. Alcohol-naïve P and NP rats display similar levels of alcohol metabolism [67]; after chronic free-choice alcohol-drinking (6–8 weeks) P rats displayed both metabolic and functional tolerance to the motor impairing and aversive effects of ethanol [68]. Moreover, similarly drinking P rats displayed withdrawal signs [69]. In addition, P rats display relapse-like drinking by exhibiting a robust alcohol deprivation effect (ADE) [70]. The ADE is a transient increase in alcohol intake after a period of ethanol withdrawal [70]. Regarding initial sensitivity, compared with NP rats, P rats are less sensitive to the ataxic [71] and hypothermic [72] effects of alcohol; and P rats develop tolerance quicker to the ataxic [71] and hypnotic effects [73]. During chronic alcohol drinking or operant self-administration, P rats achieve pharmacologically relevant blood alcohol concentrations (BACs: 80 to 250 mg%) [74,75]. These BACs parallel those observed in alcoholics. Thus, the P rat meets all criteria associated with an animal model of alcoholism [55,56,58,66].

One hundred and twelve rats were used for this study. The initial estimate of numbers of animals required for this study proposed the use of approximately 100 pairs of female rats (approximately 200 animals) using a multiple regression power analysis with an alpha of 0.001 (set low to ensure maximum sensitivity in this pilot assessment, as the true incidence in the colony was unknown) with 1 regressor and a rho^2^ value of 0.1. This calculation resulted in a projection of the need for 208 rats to result in a power of 0.9183. To reduce the overall use of animals, the experimental subjects were divided into 2 cohorts (approximately 100 animals per cohort) to perform the initial analysis. It was planned to repeat the experiment with the second cohort if no significant findings were identified in the initial cohort, but this was determined to be unnecessary.

The P selectively bred rats were an average of 6 weeks of age when the study started (range of 4 to 8 weeks of age), produced on campus from P progenitors and housed with same-sex siblings. Only females were assessed in this study as this disorder occurs more frequently in females than in males. The rats were housed within the IU animal facilities in accordance with standard operating procedures, briefly summarized here. Rats were given unrestricted access to food (Teklad Diet 7001, Envigo, Indianapolis, IN, USA) and water. Room temperatures were maintained at 21.7 +/− 1 °C and humidity was at 55 +/− 5%. Lights were maintained on a 12:12-h light:dark cycle (lights on a 0700). The rats were pair or trio housed in individually ventilated caging systems (Lab Products, Seaford, DE, USA) using standard, clear polycarbonate shoebox cages with wire lids and filter tops. Contact bedding consisting of aspen chips (Sani-Chip, PJ Murphy Forest Products, Montville, NH, USA) and paper towel nesting materials were provided to each cage.

The colony was screened quarterly by using indirect sentinels. At the time of this study, the colony was free of the following pathogens: coronavirus (sialodacryoadenitis virus), parvoviruses (NS1, rat pneumonia virus, Kilham rat virus, H1 virus, rat minute virus), theliovirus, *Clostridium piliforme*, *Mycoplasma pulmonis*, pinworms (*Aspicularis tetraptera*, *Syphacia* spp.), and fur mites (*Radfordia ensifer*, *Orinthonyssus bacoti*).

### 2.3. Assessment

Grooming behavior was assessed with a spray test that has been previously described [50]. Briefly, rats were removed from their cages and placed in a clear, Plexiglass chamber. After a minimum of 5 min within the chamber without disturbance, each rat was gently sprayed with a single application mist of water, sufficient to lightly dampen the fur on the head and shoulders. Their behavior was recorded for 15 min on digital video. This spray test was performed once for each rat. The video was scored later by a single observer (AP), and the proportion of time engaged in three grooming behaviors during the 15 min following the spray was recorded (Table 1). The videos were scored continuously with a notation of each time that each behavior was initiated. The relative frequency of each behavior was calculated by dividing the number of times each behavior was initiated by 15 min. All behavioral testing was conducted between 1200 and 1700. All data were collected within a 3-month period. Rats were identified within the cage by applying a hash mark on the tail of the second rat of each pair (rat “B”) or two hash marks on the tail of the third rat in a trio (rat “C”) with a sharpie. Rat “A” was not marked.

All cages in the colony were assessed for hair loss and skin lesions by the research staff every two weeks (and daily by the animal care staff). Tail markings were refreshed, as needed, at this time. If animals developed severe dermatitis (defined as the presence of a single open or ulcerated lesion that exceeded 2 cm in diameter), the rat was euthanized for humane reasons before the end of the study. Additionally, an animal that developed a body condition score of 2 or lower [76] was humanely euthanized. Because the study was assessing the progression of hair loss and dermatitis, treatment of ulcerative dermatitis would confound the study and its exemption was approved in advance of the study. When the rats were approximately 8 months of age, they were euthanized by carbon dioxide. Carcasses were photographed for all animals to record the presence or absence of skin lesions—those euthanized at humane endpoints and at the end of the study.

### 2.4. Statistical Analysis

The relative frequency of each of the three grooming behaviors was calculated as described above. The normality of data was determined using the Anderson–Darling test. If the data set was normal, it was analyzed with a one-way ANOVA. If the data set was not normal, it was analyzed with the Kruskal–Wallis test.

To determine if the use of the sharpie to identify animals potentially confounded the study, a chi-square test was performed to test the association between being marked with the sharpie and the presence or absence of the lesions.

To calculate the odds ratio, the grooming behaviors for oral grooming and manual grooming were set at either greater or less than 50% of the time engaged in these behaviors. Because no rat engaged in scratching behavior greater than 33% of the time during the spray test, the odds ratio was set at either zero or greater than zero for the percentage of time spent engaged in scratching behavior. In all cases, the odds ratio included the presence or absence of lesions. The odds ratio was calculated first by dividing the number of animals with lesions who had engaged in the defined proportion of the behavior by the number of animals with lesions that did not engage in the defined proportion of the behavior. This number was divided by the number of animals without lesions who had engaged in the defined proportion behavior by the number of animals without lesions that did not engage in the defined proportion of the behavior.

## 3. Results

Two rats were excluded due to technical difficulties with the recording of video for scoring and associated data loss. Out of the remaining 110 rats, 19 developed dermatitis (19/110; 17.27%). The actual numbers of animals who developed lesions with the associated relative frequency of each behavior are presented in Table 2. The use of the sharpie to identify the rats did not correspond with the development of lesions (Chi-Square = 0.718, *p* = 0.3967).

The data for the relative frequency of manual grooming and scratching behaviors were not normally distributed. The data for the relative frequency of oral grooming was normally distributed. There were no significant differences in the relative frequency of manual grooming (*p* = 0.7147) or scratching (*p* = 0.1324) in rats that developed lesions as compared to the rats that did not develop lesions using the Kruskal–Wallis test. The rats that developed lesions engaged in significantly higher relative frequencies of oral grooming as compared to those that did not develop lesions (*p* = 0.0448) using the one-way ANOVA. Data are presented in Table 3.

The amount of time spent scratching was predictive of the development of future dermatitis in the female rat with an odds ratio of 2.87 (confidence interval of 0.65, 12.73). Assuming 50% of time spent engaged in oral grooming behavior to be predictive of the development of future dermatitis in the female P rat, the odds ratio was calculated to be 1.55 (confidence interval of 0.56, 4.3). Assuming 50% of time spent in manual grooming behavior to be predictive of the development of future dermatitis in the female P rat, the odds ratio was calculated to be 0.64 (confidence interval of 0.23, 1.79).

## 4. Discussion

The hypothesis of this study was that the assessment of grooming patterns following the application of the spray test would prove predictive of the development of dermatitis in the future. The relative frequency of time engaged in the oral grooming behaviors was found to be predictive of the development of dermatitis in this cohort of rats, suggesting that this screening test is a potentially valuable tool for the implementation of proactive studies. The behavior of scratching also had a high odds ratio, suggesting that the presence of this behavior is highly predictive of the development of future dermatitis, even if the relative frequency of this behavior is not significant between rats that developed dermatitis as compared to those who did not develop dermatitis. Of note, the incidence of dermatitis reported in the cohort of animals in this study is consistent with reported incidences of dermatitis in colonies of C57BL/6J mice [77,78,79]. 

The incidence of lesion development in our population of P rats was relatively high (over 17%) as compared to the reported incidence in humans (as high as 3%) [2], but the true frequency of the disorder in humans is likely underestimated as individuals work to hide the disorder to avoid chastisement or teasing [4,8,9,10,11,12,13,14,15]. Because this stock of rat has been selected to be prone to addictive behaviors [65,66], it is possible that the incidence is higher than in the general human population, but the incidence of almost 20% provides interesting avenues for potential future research directions.

Most animal model studies of hair loss and dermatitis are therapeutic studies, evaluating potential treatments of affected humans and animals. Although treatments have value for individual humans and other animals that are affected by these body-focused repetitive disorders, the ability to identify at-risk individuals and intervene before the disorders become established would be beneficial. The spray test has been implemented to identify at-risk C57BL/6J mice and to test nutritional interventions that may prevent the onset of these body-focused repetitive disorders [50]. However, as the C57BL/6J mouse is an inbred strain of mouse, the translatability of these findings to a heterogeneous population, such as humans, may be limited. The genetic diversity of the selectively bred P rat provides a better approximation of the genetic diversity of the human population, suggesting that preventative and therapeutic studies using this animal model will result in findings that are more translatable. For example, our laboratory recently reported on how the use of nail trims can improve outcomes for ulcerative dermatitis in the P rat [80], but completion of this study required waiting for animals to develop ulcerative dermatitis prior to enrollment in the study. To complete this study, it took over 5 years, due to the relatively low incidence in the colony. The ability to screen young rats who are at risk would allow the development of a case-control study with young females with similar behavioral profiles that can be used in a study comparing potential therapeutics. 

Future directions for this model include the development of studies to test the effectiveness of therapeutic interventions (such as nutritional interventions, as has been suggested in mice [50]) in the prevention of the development of dermatitis in at-risk individuals. Genetic and epigenetic factors could be determined, providing insight into predisposing neurochemical or neurophysiological alterations observed in subjects that display or do not display TTM-like behaviors. For instance, the P rat has several neurochemical differences from their non-preferring (NP) counterparts, including serotonin and dopamine deficiencies, as well as that of associated receptors and transporters, in certain meso-corticolimbic nuclei (for a review of multiple neurotransmitter and receptor differences, see [55,65]). It is recognized that alcohol use disorders, OCD, and depression are often linked to imbalances in serotonin level and function [81,82].

## 5. Conclusions

The results of this study suggest that similar to the C57BL/6J mouse, the selectively bred P rat is a potential animal model for evaluating interventions to treat or prevent TTM behaviors. In addition to the P rat sharing clinical signs and behaviors consistent with what has been described for women affected by TTM, the spray test, and identification of rats who engage in increased scratching and oral grooming behaviors may allow for the identification of at-risk rats and the development of strategies for the prevention or early treatment of this disorder. Additional evaluation of behavioral and physiologic interactions will help to characterize the value of this model in the development of prevention and intervention strategies for TTM and other similar body-focused repetitive disorders.

## Figures and Tables

**Table 1:** Description of grooming behaviors assessed.

Grooming Type	Description
Manual Grooming (MG)	Animal grooms its muzzle, face, and head using its forepaws.
Oral Grooming (OG)	Animal grooms its body by licking, usually beginning on the upper neck and back, then extending down to more caudal areas of the body, including the tail.
Scratching (SCR)	Animal uses its hindlimbs to scratch its head, neck, and back (very fast and of short duration).

**Table 2:** Relative frequency of each behavior and the number of animals who developed lesions associated with each behavior. Data used to calculate the reported odds ratios.

Behavior	Number of Animals with Lesions	Number of Animals without Lesions
MG relative frequency less than 0.50	11	46
MG relative frequency greater than 0.50	7	46
OG relative frequency less than 0.50	8	51
OG relative frequency greater than 0.50	10	41
SCR relative frequency of 0	3	6
SCR relative frequency great than 0	15	86

**Table 3:** Mean relative frequency of rats developing lesions versus those without developing lesions. Data presented as mean +/− standard deviation.

Behavior	Animals with Lesions	Animals without Lesions
Manual grooming (MG)	0.51 +/− 0.24	0.51 +/− 0.19
Oral grooming (OG)	0.60 +/− 0.27	0.47 +/− 0.23
Scratching (SCR)	0.04 +/− 0.10	0.01 +/− 0.03

## Data Availability

The data presented in this study are available in Table 2 of this manuscript.
